# A Case of Severe Aortic Stenosis Mimicking Acute Coronary Syndrome: Diagnostic Challenges and Surgical Management

**DOI:** 10.7759/cureus.94040

**Published:** 2025-10-07

**Authors:** Waleed Abdelraheim, Mohamed Ghonem, Atef Ghoneim, Mohamed Abdelaal

**Affiliations:** 1 Cardiology, University Hospitals of Leicester NHS Trust, Leicester, GBR; 2 School of Medicine, New Giza University, Cairo, EGY

**Keywords:** bicuspid aortic valve disease, critical aortic stenosis, dilated aortic arch, infective endocarditis, ste-acs

## Abstract

Chest pain with elevated troponin and ECG changes often prompts a diagnosis of acute coronary syndrome (ACS). However, this presentation can mask other critical pathologies. We report a case of a 52-year-old male who presented with exertional chest discomfort and progressive dyspnea. Initial workup, including troponin elevation and lateral ST-segment depression, led to a presumptive diagnosis of ACS. However, further imaging revealed severe aortic stenosis (AS) secondary to a bicuspid aortic valve (BAV), with a significantly dilated ascending aorta. Intraoperatively, an annular abscess was discovered beneath the right coronary cusp, confirming a missed diagnosis of infective endocarditis (IE). The patient underwent a successful Bentall procedure with aortic root and valve replacement. This case illustrates the diagnostic challenge posed by overlapping cardiovascular pathologies and emphasizes the importance of thorough evaluation in patients with atypical ACS presentations, particularly when structural heart disease is suspected.

## Introduction

Acute coronary syndrome (ACS) is a common cause of chest pain and elevated cardiac biomarkers, typically warranting urgent antithrombotic and invasive therapy. However, in a subset of patients - estimated at 1% to 13% - coronary angiography reveals no obstructive coronary artery disease [1]. In such cases, alternative causes like severe aortic stenosis (AS) must be considered. Severe AS can lead to subendocardial ischemia due to increased myocardial oxygen demand and reduced coronary perfusion, even in the presence of normal coronary arteries, and may mimic ACS clinically [1,2]. Furthermore, elevated cardiac troponins, such as troponin I (cTnI), can occur in AS due to myocardial strain, further complicating the diagnostic process [3,4]. This case report highlights the complexity of managing a patient with very severe AS presenting with angina and elevated troponin, initially presumed to have ACS. It underscores the importance of early imaging and a multidisciplinary approach in distinguishing between true ACS and ACS mimics in structurally abnormal hearts [5].

## Case presentation

A 52-year-old man with a background of well-controlled essential hypertension presented to the emergency department with a three-day history of recurrent, non-radiating chest tightness, described as pressure-like, exertional, and rated 6/10 in intensity. He also reported progressive exertional dyspnea over the past week. Notably, he had completed a course of oral antibiotics for a recent urinary tract infection one week prior.

On physical examination, the patient was febrile (37.8°C), hypertensive (BP 140/95 mmHg), and tachycardic (HR 105 bpm), with a respiratory rate of 20 breaths per minute and oxygen saturation of 94% on room air. Auscultation of the chest revealed bilateral basal crackles, and a prominent ejection systolic murmur was best heard at the right second intercostal space.

**Figure 1:**
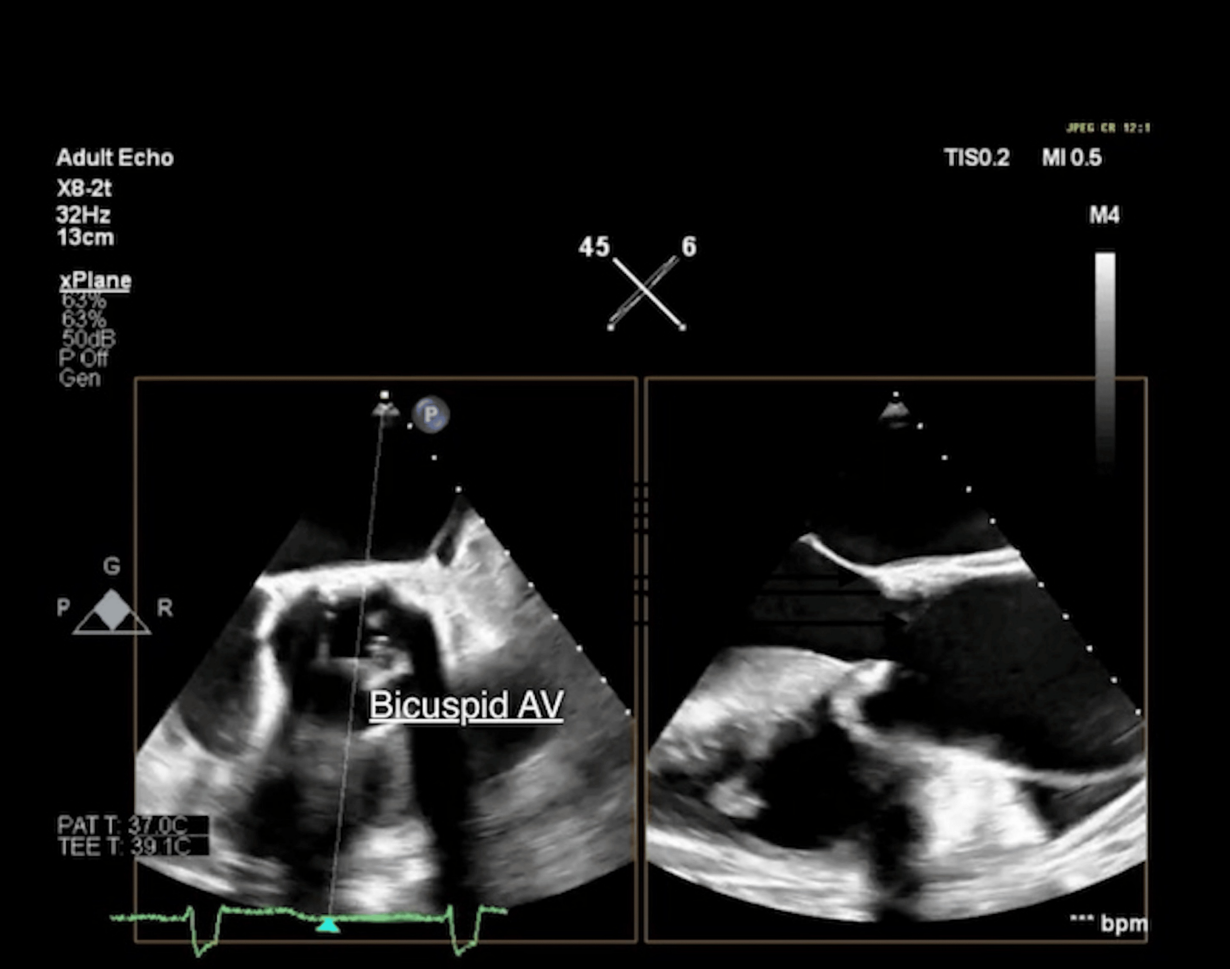
Transesophageal echocardiogram (TEE): Bicuspid aortic valve (AV) in both aortic valve short axis view (Left) and long axis view (Right).

A transesophageal echocardiogram (TEE) was subsequently carried out, confirming the presence of a BAV with severe AS (maximum velocity 5.0 m/sec), moderate aortic regurgitation (AR), impaired LV systolic function, and a markedly dilated ascending aorta (Valsalva 4.1 cm, ST junction 4.6 cm, proximal ascending aorta 4.9 cm) (Figure 1).

Blood cultures were negative. Given the structural abnormalities and hemodynamic significance of the valvular lesion, the case was discussed in a multidisciplinary team (MDT) meeting.

The differential diagnosis at presentation was broad and complex, reflecting the overlapping clinical features of several critical cardiovascular conditions. Initially, acute coronary syndrome (ACS) was the primary consideration, strongly supported by the patient's exertional chest pain, markedly elevated troponin (1938 ng/L), and lateral ST-segment depression on the ECG. However, the presence of a prominent ejection systolic murmur and progressive dyspnea raised suspicion for severe symptomatic aortic stenosis, a diagnosis subsequently confirmed by echocardiographic findings. The normal coronary arteries on angiography effectively excluded ACS and established severe AS as the primary cause of the patient's presentation. Infective endocarditis was not suspected preoperatively, given the absence of vegetations on TOE and negative blood cultures, though it was retrospectively considered after intraoperative findings. Acute aortic syndrome was suspected early due to the combination of chest pain and aortic dilatation but was later excluded by imaging.

Based on the combined findings of severe symptomatic aortic stenosis, moderate AR, BAV, impaired LV function, and ascending aortic aneurysm, the MDT recommended surgical intervention. The patient underwent a Bentall procedure consisting of composite graft replacement of the aortic valve, aortic root, and ascending aorta, with coronary artery reimplantation.

Intraoperatively, a small intimal tear was discovered in the ascending aorta. More significantly, an abscess cavity was identified beneath the annulus of the right coronary cusp, partially destroying the surrounding tissue - findings highly suggestive of previously unrecognized infective endocarditis. Despite negative preoperative blood cultures and no vegetations detected on TOE, these intraoperative findings prompted prolonged intravenous antibiotic therapy postoperatively.

The patient was commenced on flucloxacillin IV for 6 weeks and warfarin lifelong (target international normalized ratio (INR) 2.5, range 2.0-3.0). A PET scan was recommended to assess for deep-seated infection. The patient's recovery was uneventful. He was extubated successfully, transferred from the intensive care unit to the cardiology ward, and completed the prescribed antibiotic regimen. He was discharged home in a stable condition following clinical improvement and multidisciplinary follow-up.

## Discussion

This case presents a complex interplay between severe aortic stenosis (AS) and infective endocarditis (IE), initially mimicking acute coronary syndrome (ACS), and reflects several important concepts in modern cardiology practice [6]. The diagnostic and therapeutic challenges encountered underscore key pathophysiological mechanisms and management considerations in structural heart disease [7].

In severe AS, myocardial ischemia may develop even in the absence of obstructive coronary artery disease (CAD), due to several mechanisms: pressure overload-induced left ventricular hypertrophy with reduced capillary density, impaired coronary flow reserve (often reduced to 1.5-2.0 compared to the normal 3.5-5.0), and supply-demand mismatch during exertion or tachycardia [8]. These mechanisms commonly result in modest elevations in cardiac troponin levels, generally <500 ng/L [9]. In this case, however, troponin levels reached 1938 ng/L - markedly higher than typical for isolated AS - prompting consideration of a complicating factor. The subsequent diagnosis of IE offered an explanation, with several contributory mechanisms including cytokine-mediated demand ischemia (e.g., interleukin (IL)-6 and tumour necrosis factor alpha (TNF-α)), microembolic phenomena, and annular destruction affecting coronary perfusion [10].

This case also illustrates how aortic stenosis (AS) can be misdiagnosed as acute coronary syndrome (ACS). The patient presented with typical anginal chest pain, dynamic ECG changes, and markedly elevated troponin levels. This phenomenon, termed the "AS-ACS paradox", is well documented. Up to 70% of patients with severe AS report angina, and 20-30% present with ACS-like features despite having no obstructive coronary artery disease (CAD). Furthermore, troponin levels have been shown to correlate with AS severity (r = 0.62), complicating the clinical picture [1].

Echocardiography remains the cornerstone in the initial assessment of suspected AS, with transthoracic echocardiography (TTE) offering rapid evaluation of valve structure and function. In this case, TTE confirmed severe AS with preserved systolic function, but no immediate evidence of complications. Given the discordance between clinical presentation and imaging findings, coronary angiography was used to exclude coronary artery disease and assess for aortic pathology, revealing ascending aortic dilation. Ultimately, transesophageal echocardiography (TOE) confirmed the diagnosis by demonstrating detailed valve morphology. TOE has superior sensitivity for detecting perivalvular complications of IE and remains the imaging modality of choice in such cases [11].

The presence of triple pathology - severe AS, annular destruction, and aortic root dilation - necessitated early surgical intervention. The Bentall procedure, involving replacement of the aortic valve, aortic root, and ascending aorta with reimplantation of coronary arteries, was chosen. This operation is preferred in complex cases, especially those with root abscess or dilation, and offers improved outcomes compared to isolated valve replacement. Five-year survival following the Bentall procedure can reach 78%, particularly in younger patients with bicuspid aortic valves [10].

## Conclusions

This case underscores the importance of maintaining a broad differential diagnosis in patients with AS presenting with chest pain, especially when clinical and biomarker findings are disproportionate. Severe aortic stenosis can present identically to acute coronary syndrome with significant troponin elevation and ischemic ECG changes, creating substantial diagnostic challenges for clinicians. Normal coronary arteries in the setting of markedly elevated troponins should prompt immediate evaluation for structural heart disease, particularly valvular pathology.

While the incidental discovery of culture-negative infective endocarditis adds complexity to this case, the primary educational value remains demonstrating how severe AS can mimic ACS. This case highlights the critical role of advanced imaging modalities and a collaborative, multidisciplinary approach in formulating individualized management plans that deviate appropriately from standard ACS protocols when warranted by the clinical scenario.
